# *A* Case *of* Tetanus, *from a* Gun-Shot Wound, *Treated Successfully with* Laudanum *and the* Warm Bath

**Published:** 1813-02

**Authors:** 

**Affiliations:** Assistant Surgeon to H. M. 19th Regt. of Foot


*For the Medical and Physical Journal.*

### *A* CASE *of* TETANUS, *from a* GUN-SHOT WOUND, *treated successfully with* LAUDANUM *and the* WARM BATH.

*By* Mr. LEATH, *Assistant Surgeon to H. M.* 19*th Regt. of Foot.*

IN the observations on Tetanus, by Dr. Christie, of Cheltenham, which appeared in the last Number of the Edinburgh Medical and Surgical Journal, I observe a short notice of a case of Tetanus, which fell under my treatment while I was in the medical charge of the 19th regiment of foot, at Ceylon. The importance with which the attention of that gentleman stamps the case, has induced me to send you some further remarks, transcribed from my notes, together with a Table exhibiting the quantity of laudanum taken by the patient; by which, as I increased and decreased the number of doses, as the tetanic symptoms prevailed and remitted, the fluctuation of the disease may be as well understood as by a detailed narrative of the daily appearances throughout the disorder.

On the 27th of May, 1808, Francis M'Gowan, twenty-two years of age, a private in His Majesty's 19th regiment of foot, was brought into the hospital at Columbo, at ten o'clock at night, in consequence of a gun-shot wound inflicted in an attempt to commit suicide, in a fit of jealousy.

For several months, previous to the present accident, the patient had suffered greatly from dysentery, and continued at times to pass blood with his stools; yet his health and strength, for some weeks past, had been so much recovered, that he was able to perform his duties as a soldier.

From the 29th of May to the 12th of June inclusive, the wounds were at times painful and irritable, and became much enlarged by some loose pieces of bone, that protruded themselves at their orifices. On the 13th, tetanic symptoms appeared with great violence; they continued on the 14th, when the pain in his arm was so excessive, that he was anxious to undergo amputation, which, till then, he had refused to submit to; and, as I deemed it absolutely necessary, towards arresting the progress of the disease, to remove the source of such extreme irritation, as the wound had then grown, I took off the arm, about three inches above the joint of the elbow.

On examining the removed limb, it appeared that the ball had passed through the cavity of the joint, and had completely broken off the external condyle of the humerus, which it had much splintered, and detached from the body of the bone: the internal condyle was also broken off, but not detached from the bone. Nearly three-fourths of the cartridge was found lying within the cavity of the joint.

On the 15th, the tetanic symptoms continued; but, on the morning of the 16th, he was so much easier, and the violence of the tetanic symptoms so much abated, that I ordered his medicines to be given at longer intervals: the symptoms, however, increased in the evening, and I directed his doses to be exhibited throughout the night with still less interval than before. From the 16th to the 29th the symptoms continued with but few and slight remissions; and I gradually increased the laudanum to the quantity displayed by the Table.

On the morning of the 20th, in a conversation with Mr. Christie, and some more medical gentlemen, at a friend's house, great apprehensions were expressed, lest the large doses of laudanum which I had ordered might in themselves prove fatal to the patient, and leave it doubtful whether his death was to be attributed to the disease or the remedy. I was induced by these suggestions, as there now again appeared a slight remission of the symptoms, to diminish considerably (as will be seen in reference to the Table) the quantity of laudanum; but, on the return of the symptoms soon after with greater violence, I returned to my former quantity, and even increased it; yet not without great anxiety of mind, and uncertainty of the event, occasioned by the reflexion, that I was proceeding in opposition to opinions supported by the best modern authorities.

TABLE

TABLE.

| 1808. | Laudanum. | | | Sp. Æth. Nitr. | The Warm Bath. | Ung. Hydr. fort. with Camphor. |
|---|---|---|---|---|---|---|
| | ℥ | ʒ | gtt. | ʒ | | |
| June 13 | | | 160 | | | |
| 14 | | 3 | 20 | | | |
| 15 | | 7 | | | | |
| 16 | | 6 | | | | |
| 17 | 1 | | | | | |
| 18 | 2 | 4 | | 10 | | |
| 19 | 2 | 1 | | 11 | | |
| 20 | 1 | 4 | | 6 | j | |
| 21 | 2 | 3ſs | | | | |
| 22 | 2 | 6 | | | j | |
| 23 | 2 | 6 | | | j | |
| 24 | 2 | 7 | | | ij | |
| 25 | 3 | 1ſs | | | ij | |
| 26 | 3 | 4 | | | ij | ʒiij gr. xv |
| 27 | 3 | 4ſs | | | ij | ditto. |
| 28 | 3 | 4ſs | | | ij | ditto. |
| 29 | 3 | 4ſs | | | ij | ditto. |
| 30 | 3 | 4ſs | | | ij | ditto. |
| July 1 | 3 | 4ſs | | | ij | ditto. |
| 2 | 3 | 4ſs | | | ij | ʒiſs vij |
| 3 | 3 | 6 | | | ij | |
| 4 | 3 | 6 | | | ij | ʒſs |
| 5 | 3 | 6 | | | ij | |
| 6 | 3 | 6 | | | ij | |
| 7 | 3 | 6 | | | ij | |
| 8 | 3 | 6 | | | j | |
| 9 | 3 | 6 | | | j | |
| 10 | 3 | 6 | | | j | |
| 11 | 3 | 6 | | | j | |
| 12 | 3 | 6 | | | j | |
| 13 | 3 | 6 | | | | |
| 14 | 3 | | | | | |
| 15 | 1 | 7 | 40 | | | |
| 16 | 1 | 4 | | | | |
| 17 | 1 | 1 | 20 | | | |
| 18 | | 7 | | | | |
| 19 | | 5 | | | | |
| 20 | | 3ſs | | | | |
| 21 | | 2 | | | | |
| 22 | | 1ſs | | | | |
| 23 | | 1 | | | | |
| 24 | | ſs | | | | |

A very

A very similar case (published in the 20th volume of Dr. Duncan's Commentaries), treated with laudanum and the warm bath, by Dr. Mackie, of Southampton, put into my hands next morning, at once relieved me from my disquietude, and determined me to persevere in the plan which I had adopted.

From the 20th to the 26th, the increasing severity of the symptoms made me increase the laudanum to the quantity stated opposite the correspondent dates in the table, when the violence and obstinacy of the disease raised such doubts in the minds of my medical friends, that I yielded to the suggestions of Mr. Christie in having recourse to mercury; which, in the quantities also specified in the Table, was rubbed, twice a-day, into the neck, belly, and thighs, of the patient, that no medicine, supported by the authority of the most approved modern writers on tetanus, might be omitted. For the same reason I had cold water kept in readiness for the purpose of affusion, but it was never used, as the warm bath was constantly followed by some remission of the symptoms.

The nitrous spirit of ether was so nauseous to the patient, that, though I continued to prescribe it, he frequently spilled it to avoid taking it; an irregularity that gave me but little anxiety, as I did not place much dependance on that medicine. I am so uncertain of the quantity which he really swallowed, that, except for three days, I have omitted to specify it in the Table.

The mercury was continued till the 4th of July, when I left it off, no beneficial effect having been produced by it on the disease, though it had affected the mouth of the patient.

I then returned to the sole use of laudanum, increasing the quantity as the disorder increased in violence, to three ounces six drachms daily, a quantity, I believe, never before given, but which I was fully prepared even to exceed; when in a few days the disorder abated, and, on the 13th of July, was so completely subdued, that I began, and continued, to reduce the quantity of laudanum, though not so regularly as I wished, from the obstinate refusal of the patient, when he was become convalescent, to take his medicine, which he had never done in the height of the disease.

Important as I deemed it to join the use of the warm bath with the laudanum (for I am of opinion that the utility of the one depends essentially on the other, and that either would be of but little service by itself), I delayed it, under the apprehension that it might be detrimental to the stump.

The latter stages of the disease were attended with so much debility, that I made less frequent use of the warm bath, allowed allowed him to remain in it a shorter time, and soon after discontinued it altogether. The abrupt deduction from the aversion of the patient already mentioned) in the quantity of laudanum, which had supported his strength by its powerful stimulus during the progress of the disease, without doubt increased his debility, which, when the disorder left him, was so excessive, that it filled me with the most alarming apprehensions. I then indulged him in the free, but regular, use of wine, which, during the continuance of the disease, I had refused him, lest it should counteract the influence of the laudanum.

I preferred the use of laudanum to opium in a solid form: from its liquid state, it was easier of solution in the stomach, and, consequently, more speedy in its operation, its effects certain, and soon determined. I could, therefore, at short intervals, give repeated doses, and increase them in a degree sufficient to keep up at pleasure the peculiar influence which that medicine is calculated to produce on the system, and which I deemed necessary to subdue that of the disease.

Solid opium is slow in its effects, from the time taken for its dissolution, which is much longer than the intervals at which it is required to be given in this disease. It is also probable that the dissolvent powers are diminished by the opium: there must, consequently, be a quantity of that medicine at last accumulated in the stomach, and operating at once as a dose much larger than a prescriber would ever have chosen to administer at one time. I cannot, therefore, help thinking that the unhappy issue in cases of tetanus treated by opium in its solid form, is to be referred as much to the remedy as the disease.

The mercury, while it produced no good effect in itself, yet, given so largely as to affect the mouth of the patient, must, by its different and very powerful stimulus, have, in some measure, suspended the influence of the laudanum; and have, therefore, rendered a much greater quantity of the latter medicine necessary, than would otherwise have been requisite to subdue the disease.

I cannot dismiss this subject without expressing my acknowledgments for the confidence which I derived from the perusal of Dr. Mackie's case, in the mode of treatment which I had begun; and I shall feel much gratified if I should be instrumental in recalling the attention of the public to the judicious remarks with which he has prefaced his narrative, in recommendation of the means by which he was completely successful, and by which I have been so fortunate as to succeed in a case of severe tetanus, in a climate generally believed to conspire more with this terrible disease against the patient, than the milder climates of Europe.

